# Perspectives on AI-based recommendations for mask-wearing and COVID-19 vaccination for transplant recipients in the post-COVID-19 era

**DOI:** 10.1080/0886022X.2024.2337291

**Published:** 2024-04-07

**Authors:** Oscar A. Garcia Valencia, Charat Thongprayoon, Jing Miao, Jackrapong Bruminhent, Iasmina M. Craici, Wisit Cheungpasitporn

**Affiliations:** a Division of Nephrology and Hypertension, Department of Medicine, Mayo Clinic, Rochester, MN, USA; b Department of Medicine, Division of Infectious Diseases, Faculty of Medicine Ramathibodi Hospital, Mahidol University, Bangkok, Thailand; c Ramathibodi Excellence Center for Organ Transplantation, Faculty of Medicine Ramathibodi Hospital, Mahidol University, Bangkok, Thailand

**Keywords:** Artificial intelligence, transplant recipients, covid-19, Mask-Wearing recommendations, vaccine guidance

## Abstract

In the aftermath of the COVID-19 pandemic, the ongoing necessity for preventive measures such as mask-wearing and vaccination remains particularly critical for organ transplant recipients, a group highly susceptible to infections due to immunosuppressive therapy. Given that many individuals nowadays increasingly utilize Artificial Intelligence (AI), understanding AI perspectives is important. Thus, this study utilizes AI, specifically ChatGPT 4.0, to assess its perspectives in offering precise health recommendations for mask-wearing and COVID-19 vaccination tailored to this vulnerable population. Through a series of scenarios reflecting diverse environmental settings and health statuses in December 2023, we evaluated the AI’s responses to gauge its precision, adaptability, and potential biases in advising high-risk patient groups. Our findings reveal that ChatGPT 4.0 consistently recommends mask-wearing in crowded and indoor environments for transplant recipients, underscoring their elevated risk. In contrast, for settings with fewer transmission risks, such as outdoor areas where social distancing is possible, the AI suggests that mask-wearing might be less imperative. Regarding vaccination guidance, the AI strongly advocates for the COVID-19 vaccine across most scenarios for kidney transplant recipients. However, it recommends a personalized consultation with healthcare providers in cases where patients express concerns about vaccine-related side effects, demonstrating an ability to adapt recommendations based on individual health considerations. While this study provides valuable insights into the current AI perspective on these important topics, it is crucial to note that the findings do not directly reflect or influence health policy. Nevertheless, given the increasing utilization of AI in various domains, understanding AI’s viewpoints on such critical matters is essential for informed decision-making and future research.

## Introduction

In the evolving landscape of the post-COVID-19 era, two crucial public health considerations emerge for high-risk groups such as organ and kidney transplant recipients: mask-wearing and COVID-19 vaccination [1–3]. These individuals, on immunosuppressive therapy, face unique challenges due to their heightened vulnerability to infections [3]. This article demonstrated the role of artificial intelligence (AI), particularly ChatGPT 4.0, in providing recommendations on these critical aspects [4]. The aim is to evaluate the AI’s guidance on mask-wearing for both organ transplant recipients and healthy transplant providers, as well as its advice on COVID-19 vaccination for kidney transplant recipients, assessing the AI’s adaptability, potential biases, and capability to navigate complex medical decision-making.

To evaluate the efficacy and adaptability of ChatGPT 4.0 in providing health recommendations for mask-wearing and COVID-19 vaccinations specifically tailored for organ transplant recipients, we designed a structured series of scenarios. These scenarios were meticulously crafted to reflect a wide array of real-world conditions that transplant recipients might encounter, including various levels of social interaction, exposure risk, and individual health statuses.

## Scenarios development and execution

- A total of 50 unique scenarios were developed, each designed to test the AI’s response to different variables related to mask-wearing and vaccination recommendations for organ transplant recipients.
- These scenarios were categorized based on environmental settings (e.g., indoor vs. outdoor, crowded vs. sparse), vaccination status (e.g., fully vaccinated, partially vaccinated, unvaccinated), and specific health concerns (e.g., history of vaccine side effects, underlying health conditions).
- Each scenario was run through ChatGPT 4.0 three times to ensure consistency in the AI’s recommendations and to account for any variability in responses.

Prior to presenting the prompts, it’s important to note the structured design of our study:

A total of 50 unique scenarios were developed, each designed to test the AI’s response to different variables related to mask-wearing and vaccination recommendations for organ transplant recipients.

Following this setup, we engaged ChatGPT 4.0 with specific prompts tailored to elicit detailed recommendations for each scenario. The prompts provided to ChatGPT 4.0 were as follows:

For the section discussing mask-wearing recommendations, we presented ChatGPT 4.0 with the prompt:

Provide recommendations on mask-wearing for organ transplant recipients in various settings, including indoor public spaces, outdoor gatherings, and healthcare facilities.

Regarding the insights on COVID-19 vaccination, the prompt was:

Offer guidance on COVID-19 vaccination for kidney transplant recipients, considering factors such as vaccination status, potential side effects, and the timing post-transplantation.

## Evaluation criteria for recommendations

- To determine the strength of the AI’s recommendations, we employed a standardized assessment scale ranging from ‘weak’ to ‘strong’. This scale was based on the specificity, assertiveness, and context-appropriateness of the AI’s advice in each scenario.
- A ‘strong recommendation’ was classified as such if the advice provided by the AI was clear, directly applicable to the scenario at hand, and supported by relevant health guidelines or scientific evidence. For mask-wearing, a strong recommendation implied an unequivocal endorsement of mask use in settings with high transmission risk. For vaccinations, it meant an unambiguous recommendation for receiving the COVID-19 vaccine, with specific considerations given to individual health statuses and concerns.
- The determination of recommendation strength was further substantiated by a consensus among our research team members, who independently reviewed the AI’s responses to each scenario and agreed on the classification based on predefined criteria.

### Discussion on mask-wearing

**Figure 1.**
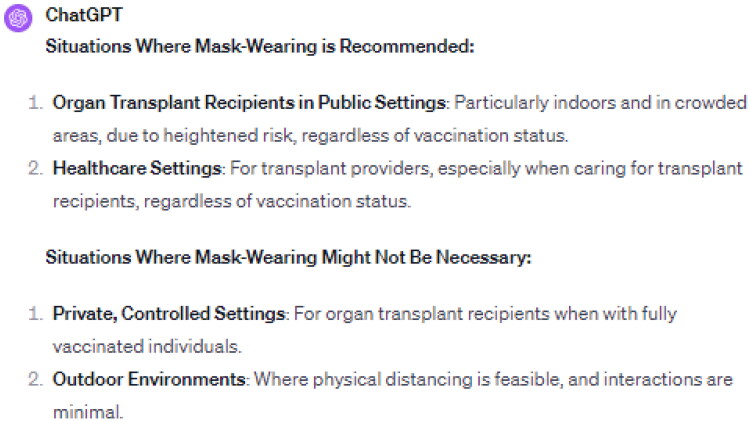
Summary of ChatGPT 4.0 Perspectives on Mask-Wearing Recommendations for Organ Transplant Recipients and Providers.

The necessity of mask-wearing in various scenarios was a significant focus of this study. AI, in this case, ChatGPT 4.0, was tasked with determining the necessity of mask-wearing in diverse settings, including crowded public areas, healthcare facilities, and private gatherings (Figure 1).

The results indicated a strong recommendation for mask-wearing among organ transplant recipients, particularly in indoor and crowded settings, acknowledging their increased risk. Conversely, in private, controlled environments with fully vaccinated individuals, and in certain outdoor settings where social distancing is feasible, the AI deemed mask-wearing less critical. For healthy transplant providers, the recommendation from AI was primarily oriented toward healthcare settings, emphasizing the need for contextual and situation-specific guidance in mask-wearing practices.

### Insights on COVID vaccination

**Figure 2.**
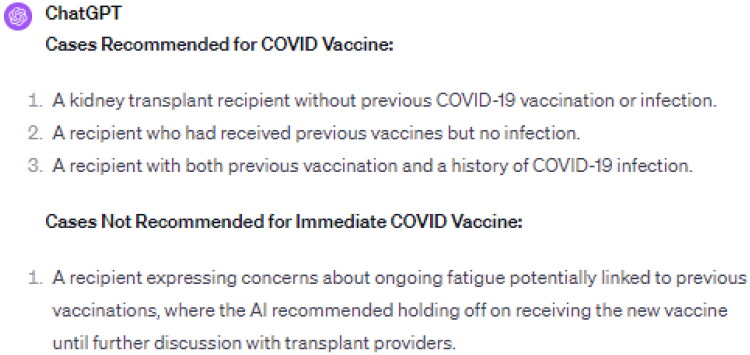
Summary of ChatGPT 4.0 Perspectives on COVID-19 Vaccination for Kidney Transplant Recipients.

Addressing the concerns about COVID-19 vaccination, particularly for kidney transplant recipients, was another key aspect of this study. Given their immunosuppressed status, these recipients’ apprehensions about vaccine side effects necessitated a careful approach. ChatGPT 4.0 was evaluated on its ability to provide tailored vaccination advice across various scenarios, including recipients with different vaccination and infection histories, and those expressing specific health concerns (Figure 2).

**Figure 3.**
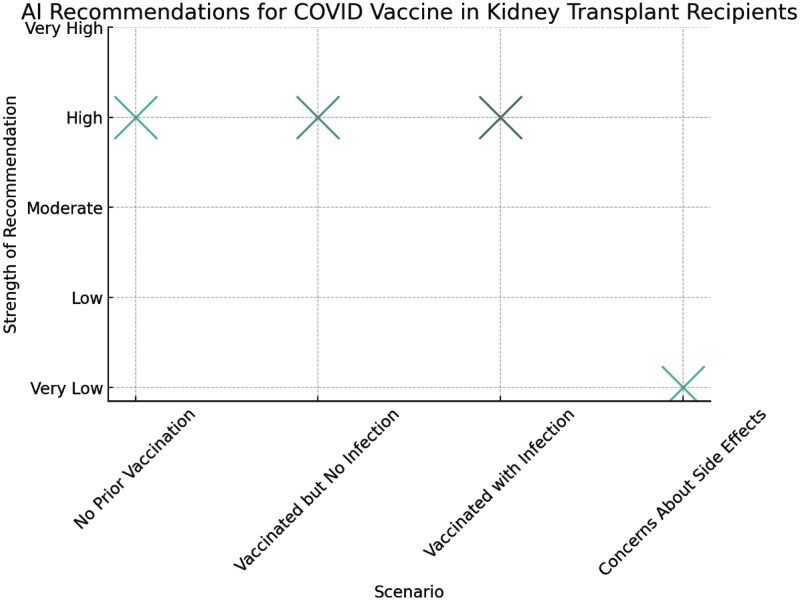
The chart visualizes the strength of ChatGPT 4.0’s perspectives across various scenarios. The vertical position indicating the strength of the recommendation.

The AI recommended the updated COVID-19 vaccine in almost all scenarios. Notably, in cases where recipients expressed concerns about ongoing fatigue potentially linked to previous vaccinations, the AI advised a more cautious approach, suggesting postponement of the vaccine until further consultation with healthcare providers (Figure 3).

### Integrated approach of AI

The study’s findings underscore ChatGPT 4.0’s comprehensive approach in addressing the critical public health measures for transplant recipients, namely mask-wearing and vaccination. The AI exhibited a balanced stance, ensuring a protective approach toward vulnerable transplant recipients while also considering individual health concerns and contexts. Notably, the AI’s approach in certain scenarios, such as recommending a delay in vaccination due to specific health concerns, indicated a level of adaptability and personalized consideration that is not commonly associated with automated systems. This capability can be linked to the AI’s sophisticated algorithms and learning capacities, which enable it to process a wide range of variables and provide context-relevant advice. Such adaptability is vital in catering to the intricate health requirements of transplant recipients. However, the study’s primary limitation lies in its sole reliance on ChatGPT 4.0, potentially limiting the breadth of AI capabilities explored [4,5]. Furthermore, the AI’s responses, grounded in programmed algorithms, might not capture the complete range of human medical decision-making intricacies. As a result, the applicability of these findings across different populations or AI systems should be approached with caution.

Our findings revealed a high degree of alignment between ChatGPT 4.0’s recommendations and the guidelines in many scenarios, particularly regarding the general advocacy for mask-wearing in high-risk settings and the endorsement of COVID-19 vaccination [1,2]. However, we also identified areas where the AI’s advice lacked the depth or specificity found in professional guidelines, such as nuanced considerations for individuals with a history of vaccine-related adverse effects or specific immunosuppressive regimens. The discrepancies and omissions uncovered through this analysis underscore the importance of augmenting AI-generated advice with expert human review, particularly for complex medical decisions involving high-risk patient groups. Our findings highlight the potential of AI as a tool for preliminary guidance, while also emphasizing the need for continuous updating and validation against authoritative health guidelines.

Interestingly, AI considered fatigue as a factor in delaying vaccination but significantly overlooked other crucial considerations, such as potential allergic reactions, the optimal timing for vaccination post-transplantation, and the appropriate vaccination timeline following a COVID-19 infection. These omissions point to a critical gap in the AI’s capability to provide comprehensive health recommendations, especially for organ transplant recipients who require carefully tailored medical advice. Addressing these gaps is essential to ensure that AI can effectively support the specific health needs of this vulnerable population. This situation emphasizes the need for AI algorithms to be developed with a more thorough integration of medical guidelines and patient-specific information, ensuring that all critical health factors are considered in the AI’s recommendations. Enhancing AI in this way would improve its utility as a supportive tool for healthcare providers, offering more reliable and complete guidance to help navigate the complex medical decisions faced by transplant recipients.

A limitation of our study is the exclusive use of ChatGPT 4.0, which, while more advanced than its predecessor ChatGPT 3.5, represents a specific snapshot of AI capabilities at the time of our research. The decision was based on ChatGPT 3.5’s documented poorer performance in answering nephrology test questions [4], leading us to conclude that its utilization for generating reliable health recommendations would be less effective. Nonetheless, this choice means our findings are limited to the capabilities of ChatGPT 4.0, and may not fully represent the potential variances in recommendations that could be offered by other AI models or future iterations of ChatGPT. As AI technology continues to evolve, the performance and reliability of these tools in healthcare applications will likely improve, necessitating ongoing evaluation to ensure the relevance and accuracy of AI-generated advice for clinical decision-making. Furthermore, conducting evaluations through a singular account during this period does not capture possible variations in AI responses over time, which could arise from updates or modifications in the training data. This specificity underscores the need for continuous assessment of AI tools in healthcare to maintain the accuracy and applicability of AI-generated recommendations amidst rapid technological advancements.

Future research should concentrate on engaging transplant societies and other healthcare organizations in a concerted effort to diversify the AI tools and participant bases used in studies. This focus is crucial for enhancing the validity and applicability of our findings. We have integrated into our manuscript a detailed discussion on the imperative for these entities to proactively refine their approach to content dissemination. It is recommended that these organizations prioritize the development and sharing of content that is accessible, interpretable, and optimized for search engines (SEO-friendly), ensuring AI algorithms can easily incorporate such information into their learning processes. This proactive content strategy is vital for improving the precision and reliability of AI-generated health recommendations, aligning them more closely with the latest medical standards and guidelines. Moreover, we stress the importance of fostering ongoing collaborations between AI developers and healthcare professionals. This partnership is essential to ensure that AI systems are continually updated with comprehensive, accurate medical information and have the capability to critically evaluate the credibility of their sources. Such collaborative efforts between transplant societies, healthcare organizations, and AI developers are fundamental in minimizing the dissemination of misinformation by AI and maximizing its utility in providing evidence-based recommendations to healthcare providers and patients. By focusing on these areas, future endeavors can further elucidate AI’s potential to support human decision-making in the nuanced field of personalized medicine, particularly for high-risk groups such as organ transplant recipients. This directed approach opens new pathways for investigating how AI can enhance the expertise of medical professionals in the post-COVID-19 healthcare landscape, signifying a pivotal evolution in the integration of AI within healthcare practices.

While our study underscores the potential of AI, specifically ChatGPT 4.0, in providing tailored health recommendations for organ transplant recipients, the necessity for caution is evident. Despite their advanced capabilities, AI technologies can sometimes produce incoherent answers or display variability in responses upon repeated questioning. This inconsistency underscores the importance of approaching AI-generated advice with a critical eye, especially in the healthcare domain where decisions directly impact patient outcomes. However, we also acknowledge that there are several strategies to mitigate the risk of AI inaccuracies, such as prompt engineering, the utilization of Retrieval-Augmented Generation (RAG) models, fine-tuning techniques, and the implementation of guardrails [6]. These methods can significantly reduce the likelihood of AI ‘hallucination’ or generating misleading information, enhancing the reliability of AI-generated advice. We stress that while AI can potentially serve as a valuable tool for supporting clinical decision-making in the future, its current use should be supplemented with expert human judgment. Incorporating these mitigative strategies ensures that healthcare providers can leverage AI’s ability to synthesize and analyze vast datasets while reducing the risks associated with its potential inaccuracies or inconsistencies. This balanced approach maximizes the benefits of AI in healthcare, fostering a more effective and safer application of these technologies in patient care

In conclusion, this study demonstrates the current AI perspective, specifically ChatGPT 4.0, in providing tailored health recommendations for transplant recipients. While it highlights the AI’s capability to adapt to individual health scenarios in post-pandemic healthcare, it also underscores the need for caution in interpreting these AI-generated recommendations. The findings pave the way for further exploration of AI in personalized healthcare, offering insights into its role in augmenting human medical expertise in the post-COVID-19 era under close supervision by human experts.
